# Enhanced versus extended preoperative antibiotic prophylaxis for retrograde intrarenal surgery in high infectious risk patients: a randomized controlled trial

**DOI:** 10.1007/s00345-025-06150-x

**Published:** 2025-12-29

**Authors:** Amr A. Elsawy, Ehab A. Nour, Adel Nabeeh, Ahmed R. EL-Nahas

**Affiliations:** Urology Department, Urology and Nephrology Center, Mansoura University, Mansoura, Egypt

**Keywords:** Retrograde intrarenal surgery, Flexible ureteroscopy, Infectious complications, Sepsis, Antibiotic prophylaxis

## Abstract

**Objectives:**

To compare enhanced (2 days) antibiotic prophylaxis (AP) versus extended (7 days) AP in terms of rate and grade of infectious complications post RIRS in high-risk patients. In addition, we aimed to identify the risk factors related to these devastating complications.

**Patients and methods:**

After institutional review board (IRB) approval, 145 patients were randomized into two groups; enhanced and extended groups. Baseline clinical, laboratory and radiological characteristics were compared between the two groups. Primary outcome was incidence of post-RIRS infectious complications. Secondary outcomes comprised length of hospital stay, stone free rate (SFR), unplanned visits and hospital readmissions. Univariate and multivariate logistic regression analyses were utilized to identify independent predictors of post-RIRS infectious complications.

**Results:**

The two study groups were comparable for preoperative baseline patients’ and stone characteristics. Asymptomatic bacteriuria, diabetes mellitus and lengthy dwelling ureteral stents were identified in 66 (45.5%), 51 (35%) and 113 (78%) patients, respectively. The rate of post-RIRS infectious complications was comparable between the two groups (36% in enhanced group and 24% in extended group, P = 0.81). There were no differences between the two groups in terms of grade of infectious complications or infected postoperative urine culture (P = 0.74, and 0.06, respectively). Length of stay, SFR and unplanned visits and hospital readmissions were statistically comparable between the two groups. On multivariate analysis, only preoperative pyuria and lengthy dwelling ureteral stents were independently associated with increased risk of post-RIRS infectious complications regardless the AP duration (relative risk 95%CI, p value = 1.49 1.22–1.83, 0.01 and 1.42 1.15–1.76, 0.02, respectively).

**Conclusions:**

Prior to RIRS in high-risk patients for infectious complications, enhanced (2 days) AP was shown comparable to extended (7 days) AP in terms of rate and grade of infectious post-operative events. Preoperative pyuria and lengthy dwelling ureteral stents were independent risk factors for post-RIRS infectious complications regardless the AP duration.

**Trial registration:**

Clinicaltrials.gov ID: NCT05384197.

## Introduction

Over the last years, retrograde intrarenal surgery (RIRS) has been proven as a safe and effective management option for upper urinary tract calculi. Therefore, we have witnessed its broadened indications and popularized utilization among endourological community [1].

Nevertheless, post-operative complications are not uncommon after RIRS including infectious complications which can range from simple urinary tract infections (UTIs) to severe systemic infections and urosepsis [2]. Given the devastating nature and life-threatening risk of these infectious complications, prompt preoperative identification of risk factors and optimal prophylaxis are highly advised [2].

Many risk factors have been attributed to the increased risk of post-operative infectious complications including preoperative influences as asymptomatic bacteriuria (ASB), prolonged indwelling ureteral stent, recent instrumentation, patient-specific factors as diabetes mellitus (DM), immunosuppression or stone factors as struvite composition [3]. Moreover, some intraoperative factors were identified to increase the risk of infection as prolonged surgical time and pressurized intrarenal irrigation [4].

Preoperative antibiotic prophylaxis (AP) has been primarily rationalized for high-risk patients for infectious complications as recommended by different panels of best practice statements for AP before urological procedures [5]. Moreover, different regimens of AP for high-risk infectious complications patients were emphasized in previous studies on other endourological procedures for stone diseases as ureteroscopy [6] and percutaneous nephrolithotomy [7].

The optimal duration of AP prior to RIRS in high-risk infectious complications patients remains undefined. Tremendous variability in clinical practice is existing as regard to the duration of AP caring the balance between the need for maximum eradication of existing organisms, and the concerns of antimicrobial-related adverse events (AEs) and drug resistance [5].

In this context, we aimed at comparing two different protocols for AP prior to RIRS for renal stones in high-risk patients for infectious complications (2 days; Enhanced protocol and 7 days; Extended protocol) in a randomized controlled trial (RCT).

## Patients and methods

After institutional review board approval from Mansoura University Research Ethics Committee (MS.22.00.2016), patients who fulfilled the inclusion criteria were invited to participate in this trial and were provided with an informed consent form. Eligibility criteria included age 18 years or older, who had renal stone with maximum diameter of 20 mm or less for whom RIRS was decided. Patients must have one or more of the following criteria to be considered high risk for infectious complications preoperative ASB (bacterial colonization in absence of symptoms and signs attributed to UTI), indwelling ureteral stent for more than 4 weeks or diabetes mellitus (DM). Patients who were unable to provide an informed consent, pregnant ladies, those with solitary kidney, chronic kidney disease (CKD) or received antibiotics within 7 days prior to enrolment were excluded from the study.

### Pre-operative assessment

Baseline laboratory investigations such as urine analysis, urine culture, serum creatinine, and random blood sugar were performed. HbA1c was assessed in all patients with a history of DM or first discovered DM by mean blood sugar at enrolment. In addition to renal ultrasonography, Computed Tomography Kidney-Ureter-Bladder (CT-KUB) was performed highlighting the stone number, location, volume (Volume = Length × Width × Depth × 0.52 [8]) and hydronephrosis.

### Randomization process and study groups

Eligible patients were randomly assigned to one of the study groups using computer-generated random tables in a 1:1 ratio. Group 1 included patient who received AP for 2 days (Enhanced protocol), while group 2 comprised patients who received AP for 7 days (Extended protocol). Operating surgeons and assessors of the outcomes were blinded to patient allocation.

In patients with positive urine culture, antibiotic of choice was decided according to susceptibility results and patient tolerability with the last day of AP course being one day prior to intervention. On the other hand, patients with negative urine culture received oral Sulfamethoxazole-Trimethoprim (TMP-SMX) 800/160 mg twice daily (selected based on our institutional antibiotic stewardship program). In patients with allergy or resistance to TMP-SMX, the following antibiotics were considered in the following order: 100 mg Nitrofurantoin twice daily, 500 mg Ciprofloxacin twice daily or 200 mg Cefpodoxime twice daily.

### Procedure

All procedures were done by expert endourologists. An 11/13 Fr ureteral access sheath was introduced according to surgeon preference, and an 8.6 Fr single-use digital flexible ureteroscope (WiScope®, OTU Medical, Union City, California, USA) was utilized for all patients. Manual irrigation assisted by the scrubbing nurse was utilized in all cases, with Laser lithotripsy performed with a 30-Watt Holmium: Yttrium Aluminium Garnet (YAG) machine. Stone dusting or fragmentation was decided by the surgeon. At the end of procedure, either an external ureteral catheter or an internal dwelling double J stent was inserted according to surgeon preference and removed as per protocol (External ureteral stent at first post-operative day and double J stent after two weeks in the outpatient clinic under local anaesthesia).

### Post-procedural assessment and follow up

Postoperatively, all patients stayed in hospital for overnight observation. Monitoring for any symptoms/signs suggestive for infectious or other complications was ensured by blinded clinicians to the deployed antibiotic regimen. Suspected patients for post-operative infectious complications were assessed by urine analysis, urine culture, CBC, C reactive protein (CRP) ± blood culture (in febrile patients). Postoperative antibiotics were only utilized in those patients who developed infectious events. As per patient condition, they were discharged from the hospital and were scheduled for follow up appointment after 4-weeks for assessment of residual stones by CT-KUB.

### Outcome measures

Primary outcome was the incidence of postoperative infectious complications (defined as ≥ grade 2 according to Infection Severity Scale by the European Association of Urology (EAU) (Grade 1: cystitis—dysuria, urgency and/or suprapubic pain; Grade 2: mild to moderate pyelonephritis—temperature > 38.5C, flank pain; Grade 3: severe pyelonephritis—grade 2 + nausea or vomiting; Grade 4: urosepsis; Grade 5: severe urosepsis—grade 4 and organ dysfunction; Grade 6: uroseptic shock) [9].

Secondary outcomes included the rate of postoperative positive urine culture, length of hospital stay, stone free rate (SFR) at 4 weeks, unplanned visits and hospital readmission. Stone free status was defined as no residual fragments at follow up CT-KUB after 4 weeks.

### Sample size calculation and statistical analysis

Being our study the first randomized trial to explore the incidence of post-RIRS infectious complications in high-risk infectious patients, we proposed a sample size of 70 patients in each group to achieve a statistical power of 80% at 5% type 1 error allowing for a 10% dropout rate and 20% difference in postoperative infectious complications between both groups.

All data were computed using a commercial program "SPSS v21" (Chicago, Ill, USA). Categorical variables were compared using Chi-Square test. Continuous variables were compared using t-test. Both univariate and multivariate logistic regression analysis were utilized for the primary outcome assessment.

## Results

### Baseline demographics

**Fig. 1:**
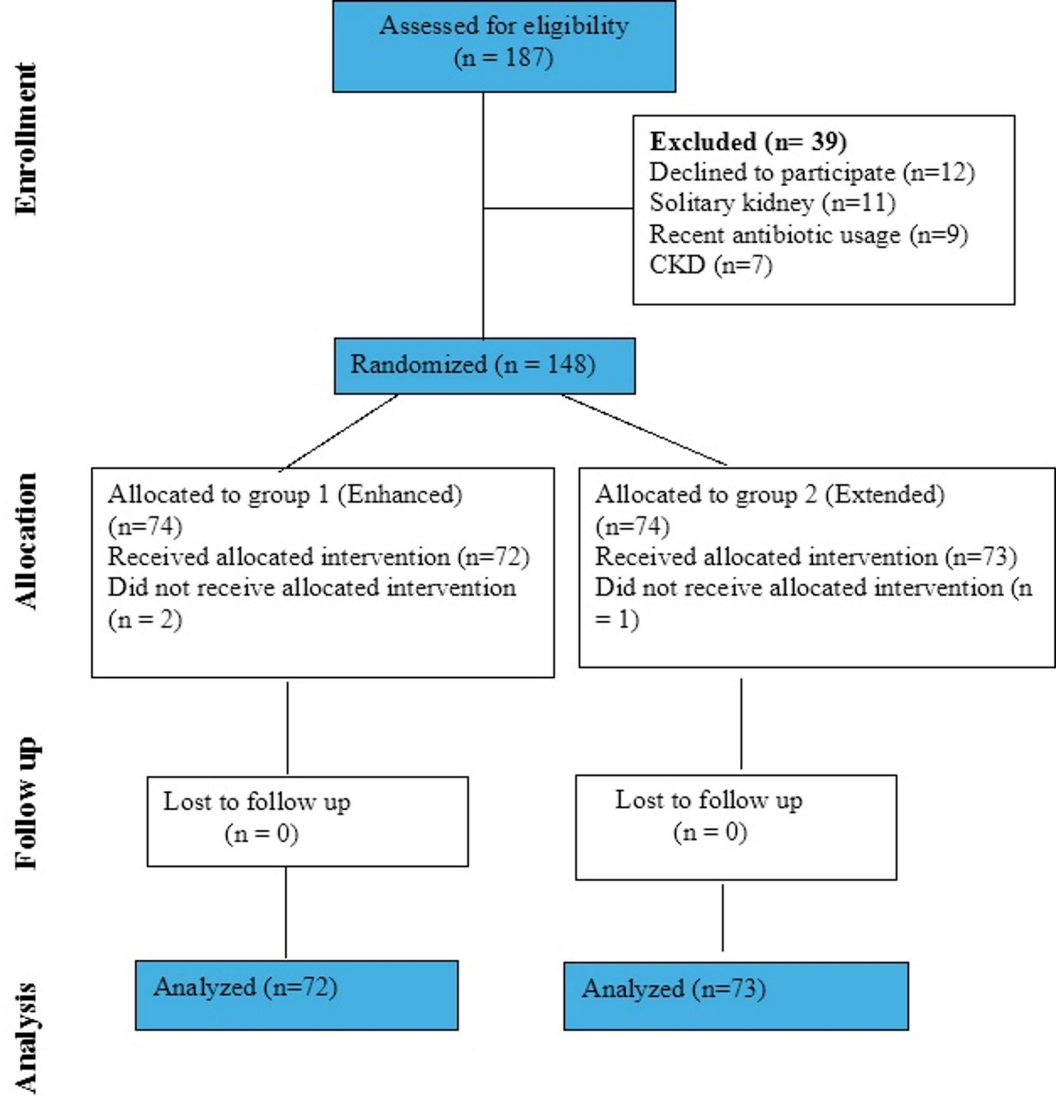
Consort flowchart of study participants

In the period between May 2022 and January 2025, 148 patients were randomly allocated to the two study groups. As shown in the study flowchart (Fig. 1), 145 (72 in enhanced group and 73 in extended group) patients were included in the final analysis.

As illustrated in Table 1, all baseline patients’ demographics and stone characteristics were comparable between the two study groups apart from preoperative hydronephrosis which was more noted in the enhanced group. Notably, all patients with preoperative hydronephrosis in both groups were mild degree and related to impacted stone at pelvi-ureteral junction.

**Table 1:** Comparison of baseline patients’ demographics, stone characteristics and operative data of the two study groups

Variable	Group 1 (Enhanced) 72 Patients	Group 2 (Extended) 73 Patients	P value
Patients’ demographics			
Gender: N (%)			0.941
Male	32 (44.4)	32 (43.8)	
Female	40 (55.6)	41 (56.2)	
Age (years): Mean ± SD	51.6 ± 11.7	54.9 ± 12.2	0.101
Diabetes: N (%)			0.201
No	43 (59.7)	51 (69.9)	
Yes	29 (40.3)	22 (30.1)	
History of Stone Treatment: N (%)			0.087
No	25 (34.7)	16 (21.9)	
Yes	47 (65.3)	57 (78.1)	
Lengthy Dwelling Ureteric Stent: N (%)			0.398
No	18 (25)	14 (19.2)	
Yes	54 (75)	59 (80.8)	
Urine Culture: N (%)			0.208
Sterile	43 (59.7)	36 (49.3)	
Infected	29 (40.3)	37 (50.7)	
Urine organism: N (%)			0.445
*Escherichia Coli*	17 (23.6)	22 (30.2)	
*Klebsiella Pneumoniae*	8 (11.1)	9 (12.3)	
*Enterococcus faecalis*	0 (0)	3 (4.1)	
*Enterobacter Cloacae*	2 (2.8)	0 (0)	
*Pseudomonas Aeruginosa*	0 (0)	1 (1.4)	
*Proteus Mirabilis*	1 (1.4)	1 (1.4)	
*Staphylococcus aureus*	1 (1.4)	1 (1.4)	
Preoperative Creatinine (mg/dl): Mean ± SD	1.1 ± 0.5	1.1 ± 0.48	0.278
Preoperative Hydronephrosis: N (%)			0.026
Absent	50 (69.4)	62 (84.9)	
Present	22 (30.6)	11 (15.1)	
Stone characteristics			
Stone Number: N (%)			0.217
One	41 (56.9)	31 (42.5)	
Two	15 (20.8)	21 (28.8)	
Three	16 (22.2)	21 (28.8)	
Stone volume (mm3): Median (IQR)	523 (297–757)	586 (391–1151)	0.076
Stone Hounsfield unit: Mean ± SD	745 ± 360	780 ± 314	0.537
Operative data			
Ureteral Access Sheath: N (%)			0.015
Yes	34 (47.2)	49 (67.1)	
No	38 (52.8)	24 (32.9)	
Laser Setting: N (%)			0.448
Dusting	42 (58.3)	47 (64.4)	
Fragmentation	8 (11.1)	4 (5.5)	
Combination	22 (30.6)	22 (30.1)	
Volume of Irrigation (Liters): Mean ± SD	5.3 ± 1.9	5.2 ± 1.5	0.874
Postoperative Drainage: N (%)			0.133
Ureteric Catheter	21 (29.2)	30 (41)	
DJ Stent	51 (70.8)	43 (59)	
Operative Time: (minutes): Mean ± SD	55 ± 23	61 ± 19	0.052

There were no statistically significant differences between the two study in the terms of Laser lithotripsy strategy, irrigation volume and operative time. Nevertheless, ureteral access sheath utilization was more noted in extended group (Table 1).

### Outcome measures

The infectious complications incidence was statically comparable between the two study groups (36% in enhanced group and 24% in extended group, P = 0.81) (Table 2). As well, there was no difference between the two groups in terms of grade of infectious complications or positive postoperative urine culture (P = 0.74, 0.06, respectively). Similarly, severe infectious complications (grade 4 or more) in the two groups were identified in about 13 patients in each group (Table 2).

**Table 2:** Comparison of the outcomes between the two study groups

Outcome	Group 1 (Enhanced) 72 Patients	Group 2 (Extended) 73 Patients	P Value
Infection Complications: N (%)			0.814
Yes	26 (36.1)	25 (34.2)	
No	46 (63.9)	48 (65.8)	
Infection Grade: N (%)			0.743
Grade 2	13 (18)	11 (15)	
Grade 3	0 (0)	1 (1.4)	
Grade 4	12 (16.7)	10 (13.7)	
Grade 5	0 (0)	1 (1.4)	
Grade 6	1 (1.4)	2 (2.7)	
Postoperative urine culture: N (%)			0.06
Infected	7 (9.7)	16 (21.9)	
Sterile	65 (90.3)	57 (78.1)	
Urine organism: N (%)			
*Escherichia coli*	3 (4.2)	7 (9.6)	0.15
*Klebsiella pneumoniae*	4(5.6)	3 (4.1)	
*Pseudomonas aeruginosa*	0(0)	4 (5.5)	
*Enterococcus faecalis*	0 (0)	1 (1.4)	
*Serratia marcescens*	0 (0)	1 (1.4)	
Length of hospital stay (day): Mean ± SD	1.9 ± 0.2	1.9 ± 0.3	0.9
Stone Free Rate: N (%)			0.4
Yes	60 (83.3)	57 (78.1)	
No	12 (16.7)	16 (21.9)	
Auxiliary Procedures and retreatment: N (%)			0.35
Inapplicable	60 (83.3)	57 (78.1)	
SWL	4 (5.6)	1 (1.4)	
2nd session FURS	5 (6.9)	9 (12.3)	
Alkalinization therapy	3 (4.2)	6 (8.2)	
Unplanned Visits: N (%)			0.8
Yes	9 (12.5)	10 (13.7)	
No	63 (87.5)	63 (86.3)	
Hospital readmission: N (%)			0.9
Yes	1 (1.4)	0	
No	71 (98.6)	73 (100)	

On the other hand, length of hospital stay, SFR at 4 weeks, need for auxiliary procedure, retreatment, unplanned visits and hospital readmissions were comparable between the two study groups (P = 0.9, 0.4, 0.45, 0.8, 0.9, respectively).

As shown in Table 3, among study participants, infectious complications were identified in 51 (26 in enhanced and 25 in extended group) patients. On univariate and multivariate logistic regression analysis, the duration of preoperative AP did not significantly increase the risk of infectious complications postoperatively (P = 0.8). It was shown that only preoperative dwelling ureteral stents more than 4 weeks and preoperative pyuria were the only independent predictors of post-operative infectious complications (relative risk, 95%CI, and P values 1.49 1.22–1.83, 0.01 and 1.42 1.15–1.76, 0.02, respectively) (Table 3).

**Table 3:** Univariate and multivariate logistic regression analysis for predictors of infectious complications

	Postoperative Infectious Complications		Univariate analysis	Multivariate analysis		
	Yes (51 patients)	No (94 Patients)	P value	RR	95% CI	P value
Gender: N (%)			0.602			
Male	24 (47)	40 (53.2)				
Female	27 (53)	54 (46.8)				
Age (year) Mean ± SD	55.5 ± 11.8	52 ± 12	0.11			
Diabetes: N (%)			0.15			
Yes	14 (27.5)	37 (39.4)				
No	37 (72.5)	57 (60.6)				
Dwelling Ureteric Stent > 4 weeks: N (%)			0.009	1.49	1.23–1.83	0.01
Present	46 (90.2)	67 (71.3)				
Absent	5 (9.8)	27 (28.7)				
Prior Stone Treatment: N (%)			0.5			
Yes	38 (74.5)	66 (70.2)				
No	13 (25.5)	28 (29.8)				
Preoperative Pyuria: N (%)			0.004	1.42	1.15–1.76	0.02
Present	48 (94.1)	70 (74.5)				
Absent	3 (5.9)	24 (25.5)				
Preoperative Urine culture: N (%)			0.5			
Present	25 (49)	41 (43.6)				
Absent	26 (51)	53 (56.4)				
Stone Number: N (%)			0.56			
One	25 (49)	47 (50)				
Two	15 (29.4)	21 (22.3)				
Three or More	11 (21.6)	26 (27.7)				
Stone volume (mm^3^) Median (IQR)	527 (358–885)	573 (301–894)	0.86			
Stone density (HU) Mean ± SD	743.3 ± 349.8	772.9 ± 331	0.6			
Hydronephrosis: N (%)			0.5			
Present	13 (23.5)	20 (21.3)				
Absent	38 (74.5)	74 (78.7)				
Duration of preoperative antibiotic prophylaxis: N (%)			0.8			
2-day (Enhanced)	26 (51)	46 (48.9)				
7-day (Extended)	25 (49)	48 (51.1)				
Preoperative antibiotic utilized: N (%)			0.9			
SMT-TMP	31 (60.8)	59 (62.8)				
Nitrofurantoin	6 (11.8)	12 (12.8)				
Ciprofloxacin	5 (9.8)	7 (7.4)				
Meropenem	8 (15.6)	14 (14.9)				
Cefepime	1 (2)	2 (2.1)				
Ureteral access sheath: N (%)			0.9			
Yes	29 (56.9)	54 (57.4)				
No	22 (43.1)	40 (42.6)				
Method of Irrigation: N (%)			0.49			
Gravity	0 (0)	2 (2.1)				
Manual	17 (33.3)	35 (37.2)				
Both	34 (66.7)	57 (60.6)				
Volume of irrigation (Litre) Mean ± SD	5.6 ± 1.6	5.07 ± 1.7	0.09			
Laser Setting: N (%)			0.6			
Dusting	33 (64.7)	56 (59.6)				
Fragmentation	5 (9.8)	7 (7.4)				
Both	13 (25.5)	31 (33)				
Laser Setting: N (%)						
Operative time (minutes) Mean ± SD	59.7 ± 18	56.4 ± 23	0.36			
Postoperative drainage: N (%)			0.6			
Ureteric catheter	19 (37.3)	32 (34)				
Double J stent	32 (62.7)	62 (66)				

## Discussion

With the growing advancement in Laser technology and flexible ureteroscopes, RIRS is gaining more popularity and number of procedures are mounting speedily. Nevertheless, despite its advantages, RIRS is associated with a worrisome risk of infectious complications. They range from postoperative fever and simple UTI to severe inflammatory response as urosepsis, which can lead to increased morbidity, patient dissatisfaction, lengthy hospital stay and mortality in severe cases [2].

The incidence of infectious complications after RIRS ranges from 2 to 40% in different reports with noted wide variability due to underreporting and non-standardized classification of infectious events post-operatively [4]. Akgul et al. has reported post-operative febrile UTI in 61 (36.3%) of 168 patients who underwent RIRS [10]. On the other hand, a lower rate of infectious complications was highlighted by Zhang and colleagues who identified only 7% rate of infectious complications in their cohort [11].

The risk of infectious complications post RIRS is influenced by many factors including patient’s morbidity, immunocompetency, preoperative bacteriuria, lengthy dwelling ureteral stents and compromised renal function [4]. As well, some operative findings, as prolonged surgical time and pressurized irrigation, were shown to be associated with increased risk of postoperative UTI and urosepsis [12].

Therefore, preoperative measures are paramount steps in reducing the risk of infectious consequences associated with RIRS by dedicated identification of high-risk patients and optimization of any existing modifiable risk factors preoperatively [13]. Current guidelines do not provide clear recommendations as regard to the optimal duration of AP prior RIRS [14]. Interestingly, the AP regimen prior to RIRS in high-risk infectious complications patients remains undefined. Widely variable clinical practice is existing as regard to the duration of AP with inclination towards shorter duration protocols aiming at minimizing antimicrobial-related AEs, resistance prevention and cost saving [5].

In our RCT evaluating high-risk infectious patients undergoing RIRS for renal stones, we found that both enhanced (2-day) and extended (7-day) protocols of preoperative AP were equivalent in term of rate and grade of infectious complications after the procedure. This finding could be helpful for standardization of the AP strategy before RIRS among practitioners. Furthermore, this can help in reducing the risk associated with antimicrobial over usage: side effects as Clostridium difficile infection [15] and multidrug resistance organisms [16].

In the present study, we have adapted the EAU Infection Severity Scale in reporting and grading the infectious complications which provides a comprehensive (both subjective and objective) clinically-relevant validated tool for infectious complications [9]. Grade 2 and 3 complications compromised febrile UTI (pyelonephritis) complications, and it was equally reported in the two study groups: 13 (17.8%) and 12 (16.5%) patients in the enhanced and extended groups, respectively. Similarly, grades 4–5 infectious complications included urosepsis and it were identified in comparable rates among the two groups: 12 (16.6%) and 11(15%) patients, in the enhanced and extended groups, respectively.

We recognized that patients with ASB, experienced equivalent incidence and grades of infectious complications regardless the duration of AP. Additionally, the causative organisms were not shown to significantly implicate the infectious events post-operatively. Similar findings were highlighted by Perez et al. who demonstrated that microbiological background did not signify the risk of post-operative infections after ureterorenoscopy [17]. Nevertheless, preoperative pyuria was shown to significantly increase the risk of infectious complications in the study groups. This observation copes with the findings of Mitsuzuka et al. and Chen et al. who identified preoperative pyuria as a risk factor for infectious complications after ureteroscopy and percutaneous nephrolithotomy, respectively [18, 19].

Our results showed that patients with dwelling ureteral stents more than 4 weeks are at higher risk for infectious complications post RIRS despite the duration of AP and urine culture preoperatively. Nevo et al. has shown the parallel findings of significant association between prolonged stent dwelling time and post-ureteroscopy time [20]. This could be hypothesized by developed bacterial biofilms in lengthy dwelling stents making it difficult for AP to eliminate it whatever the duration of treatment [21].

Different studies have demonstrated association between DM and infectious complications post RIRS [3] due to hypothesized suppressed phagocyte and granulocyte functions and impaired immune response [22]. In comparison to Li et al. who found that DM as a strong predictor of post-RIRS urinary sepsis [23], we recognised that when AP was utilized prophylactically in diabetic patients preoperatively, infectious complications were markedly reduced.

The relation between stone factors (stone burden and multiplicity) and post-RIRS infections, were thoroughly assessed in multiple studies. Larger stone volume would be associated with longer operative time and increased irrigation pressure with subsequent risk of infectious complications [24]. In addition, associated incomplete stone clearance may be attributed to the increased risk of infectious complications [24]. On the contrary, in our study, stone factors (volume, density, and multiplicity) were not identified as significant risk factors for infectious complications which could be justified by the strict inclusion criterion of stone size (20 mm or less).

Despite many operative factors as ureteral access sheath utilization, method and volume of irrigation and operative time were identified in previous reports as significant predictors of post-RIRS fever and infectious complication [3]. Our results did not show any significant impact of these parameters on post-procedural infectious events. This could be justified by either added prophylactic feature of AP or the included stone size (20 mm or less which means average operative time and limited intrarenal pressure).

Our study is acknowledged to be the first study to highlight the need for implementation of unified evidence-based practice regarding the duration of AP for patients undergoing RIRS based on patient-risk classification. Nevertheless, some limitations could be admitted. Firstly, inclusion of different risk factors (ASB, lengthy dwelling stent, and DM) in our study may confound the analysis, despite, we can argue that this heterogenicity expands the study’s generalizability, mimicking “real practice” scenarios. Secondly, lack of stone analysis, stone culture, stent culture in presented patients, may compromise the antimicrobial data which could better correlate pathogens with postoperative infections. Moreover, the variation in stent removal timing and antibiotic sensitivity patterns may influence outcomes. The relatively small sample size may limit the detection of rare infectious events. Lastly, limited stone burden in our study did not match with current practice in RIRS with expanded stone treatment and its impact on operative time, intrarenal pressure and risk of infections.

## Conclusions

Prior to RIRS in high-risk patients for infectious complications, enhanced (2 days) AP was shown comparable to extended (7 days) AP in terms of incidence and grade of infectious postoperative events. Preoperative pyuria and lengthy dwelling ureteral stents were independent risk factors for post-RIRS infectious complications regardless the AP duration. Accurate risk categorization prior to RIRS and optimal AP are highly advisable to minimize the risk of infectious encounters postoperatively while avoiding overtreatment and side effects of antimicrobials. Future multicenter studies with larger sample sizes emphasizing infectious risk categorization prior to RIRS and its prophylaxis will be informative and helpful for a safer and effective stone treatment practice.

## Data Availability

Data is provided within the manuscript.
